# On Hokusai's *Great wave off Kanagawa*: localization, linearity and a rogue wave in sub-Antarctic waters

**DOI:** 10.1098/rsnr.2012.0066

**Published:** 2013-03-06

**Authors:** J. M. Dudley, V. Sarano, F. Dias

**Affiliations:** 1 Institut FEMTO-ST, UMR 6174 CNRS-Université de Franche-Comté, 25000 Besançon, France; 2 Association Longitude 181 Nature, 12 rue la Fontaine, 26000 Valence, France; 3 School of Mathematical Sciences, University College Dublin, Belfield, Dublin 4, Ireland

**Keywords:** *Great wave of Kanagawa*, rogue waves, wave breaking, Hokusai

## Abstract

The Hokusai woodcut entitled *The great wave off Kanagawa* has been interpreted as an unusually large storm wave, likely to be classed as a rogue wave, and possibly generated from nonlinear wave dynamics (J. H. E. Cartwright and H. Nakamura, *Notes Rec. R. Soc.* **63**, 119–135 (2009)). In this paper, we present a complementary discussion of this hypothesis, discussing in particular how linear and nonlinear mechanisms can both contribute to the emergence of rogue wave events. By making reference to the *Great wave*'s simultaneous transverse and longitudinal localization, we show that the purely linear mechanism of directional focusing also predicts characteristics consistent with those of the *Great wave*. In addition, we discuss the properties of a particular rogue wave photographed on the open ocean in sub-Antarctic waters, which shows two-dimensional localization and breaking dynamics remarkably similar to Hokusai's depiction in the woodcut.

*The great wave off Kanagawa* is arguably the most famous of the many woodblock prints made by the Japanese artist Hokusai. The print (shown in figure 1*a*) shows an enormous wave on the point of breaking over cargo boats that are being sculled against the direction of the wave's travel. The wave itself is often assumed to be associated with a tsunami but, as examined in detail by Cartwright and Nakamura in this journal,^1^ the Hokusai print is far more likely to show an extreme plunging breaker, a large storm wave on the point of breaking over the approaching boats. The location of the wave is estimated to be 3 km offshore within Tokyo Bay, and the estimated wave height of around 10 m leads to the conclusion that this would be a wave of exceptionally large amplitude for this area and would be likely to fall into the category of a rogue or freak wave.

**Figure 1.**
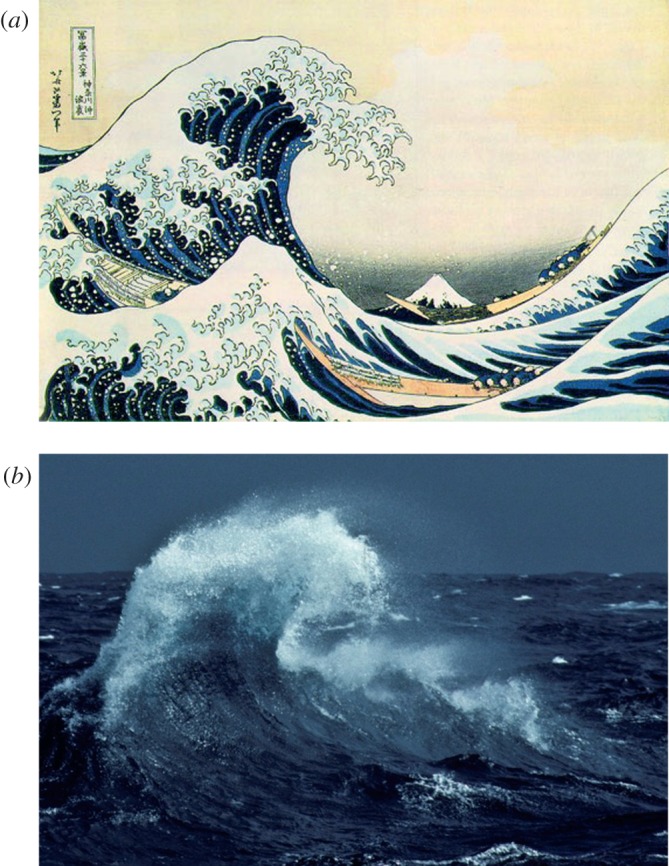
Comparison of Hokusai's *Great wave* with an observation in sub-Antarctic water. (*a*) *The great wave off Kanagawa* (*Kanagawa-oki nami-ura*) woodcut by Katsushika Hokusai. (*b*) Photograph of a breaking wave in the sub-Antarctic waters of the Southern Ocean taken from the French research vessel *Astrolabe* during one of its regular voyages between Hobart and the Dumont d'Urville Station in Adélie Land. Note the transverse and longitudinal localization of the wave, which is remarkably similar to that depicted by Hokusai. (Photograph taken by V. Sarano in 1991.) (Online version in colour.)

Rogue waves are large-amplitude surface waves that represent wave-height outliers for a particular sea state. This means that they possess height and steepness much larger than the average values of other ocean waves in the surrounding environment. It is important to distinguish rogue waves from tsunamis.^2^ A rogue wave is a strongly localized wave in space and time on the ocean's surface, whereas a tsunami is caused by massive water displacement. After their initial stage of generation, tsunamis propagate at high speed over long distances at small amplitude, typically as low as tens of centimetres on the open ocean, where they are generally unnoticed. Tsunamis only become destructive as they run up to the shore and grow to great height as the water depth decreases.

The dynamics of tsunamis is very well understood: they propagate as linear waves on the ocean^3^ until, at a distance from shore of about one-seventh of their wavelength, some nonlinear processes may have a role.^4^ In contrast, a consensus concerning the physics of oceanic rogue waves is only just beginning to be developed. Rogue waves are now generally considered to form a distinct class of ocean waves, but it seems likely that they arise from a variety of different physical mechanisms.^5^ In particular, both linear and nonlinear effects have been shown to lead to unusual wave amplification under many different conditions. For example, linear effects that can generate rogue waves include spatial focusing due to refraction in the presence of varying topography, wave–current interactions, dispersive focusing of a chirped wave train, and directional focusing of multiple wave trains. Nonlinear effects have also received much attention, especially the process of exponential amplification of random surface noise through modulation instability, and the development of large-amplitude coherent structures and related soliton dynamics. Note that whether the generating mechanism is linear or nonlinear does not enter into the definition of a rogue wave; the only criterion is whether the wave is statistically much larger than the other waves in the immediate environment. Reviews of the subject of rogue waves have been published previously.^5–7^

A possible generation mechanism for Hokusai's *Great wave* in terms of nonlinear soliton collisions was discussed by Cartwright & Nakamura^1^ and, indeed, the important role of nonlinear dynamics in wave amplification has been confirmed in several recent studies.^8–10^ However, it is also important to consider the role of linear effects in extreme wave localization, because the initial stage of wave propagation will generally begin in the linear regime, and nonlinear effects will become important only as the wave amplitude increases to an appropriate level. In the case of the *Great wave*, a particular clue to how linear effects may play a role is seen by noting the *Great wave*'s localization, both along its direction of travel and transversely—we see the wave rising from the foreground and ending in the middle ground of the print.^1^ Although such localization has been studied in the framework of nonlinear effects,^11,12^ we discuss below how it can also arise from linear propagation.

Before our discussion of this mechanism, however, we first make the important observation that such two-dimensionally localized waves do in fact occur in nature, and can assume large amplitudes and undergo wave breaking as illustrated in the woodcut. In this context we remark that although breaking waves are often associated with the laterally extended surf waves arriving on a beach, any water wave can break when the wave crest steepens sufficiently.^13^ There is nothing unphysical about a localized wave undergoing breaking at high steepness.

To confirm this explicitly, we show a photograph in figure 1*b* of an ocean wave with properties remarkably similar to Hokusai's *Great wave*. The wave is localized in the same manner, is also on the point of breaking, and we also clearly see the dramatic forward ejection of foam. This photograph is significant in that it shows the simultaneous presence of transverse localization and wave breaking on the open sea far from the shore. This provides further confirmation of the accuracy of Hokusai's depiction of nature. The photograph itself was taken in February 1991 in the sub-Antarctic waters of the Southern Ocean from the French research vessel *Astrolabe* during one of its regular voyages between Hobart and the Dumont d'Urville Station in Adélie Land. Note that the dimensions of the wave in figure 1*b* are smaller than that of the great wave in the woodcut: the wave height in the *Astrolabe* photo is estimated at 6–8 m. The height estimation was based on reference relative to the known height of the *Astrolabe*'s helipad deck, which was the point of observation. The waves on the background sea were estimated to have much lower heights, in the range 1–3 m, consistent with the interpretation of the isolated larger-amplitude wave as a rogue wave. We also note that the difference in absolute heights between this sub-Antarctic wave and the *Great wave* is unimportant for our discussion here because the essential physics of ocean wave breaking remains the same for waves over a large range of amplitudes.^13^

We now consider how such dramatic localization as seen in both this photograph and the *Great wave* woodcut may arise from linear propagation effects. Specifically, we examine the process of directional focusing, which arises whereby periodic wave trains with different directions and phases interfere together at a particular point in space and time to create an extreme rogue wave at their focus. Typical results of numerical modelling are shown in figure 2.^14^ In fact, the modelling is based on fully realistic propagation equations that include both linear and nonlinear effects, but the essential concentration of energy at the point of focus arises from linear convergence to a focus. Nonlinearity has a dominant role only as the wave approaches the linear focus, where it increases the wave steepness to the point of breaking. The visual similarity of the numerical modelling of directional focusing to the localization properties seen in the woodcut is immediately apparent, and thus directional focusing is clearly also a mechanism that could underlie the formation of the *Great wave*.

**Figure 2.**
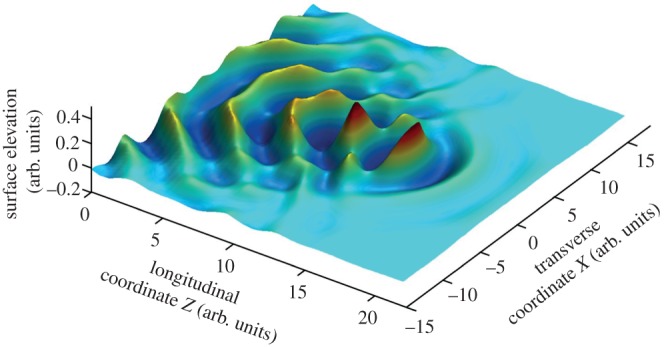
Numerical results showing directional focusing of periodic wave trains towards an extreme wave at the focus. The wave steepness at this point is such that we can clearly see the wave crest overturning to the point of breaking. (Adapted from Fochesato *et al.*^14^) (Online version in colour.)

At this point we can note another feature of the *Great wave* that suggests the presence of several surrounding waves of large amplitude. Although these other waves appear outside the frame of the image, it is natural to consider whether the fishermen are actually experiencing a ‘wave packet’ of several large rogue waves. This is in fact perfectly plausible. Although each single rogue wave crest is localized in space and time, numerical studies and observation have also reported that several (typically two or three) consecutive rogue waves can be generated.^7,15,16^ The occurrence of multiple rogue waves in this way can be caused by nonlinear effects, but is also possible with directional focusing.^14^

Our conclusions concerning *The great wave off Kanagawa* can be easily stated. When discussing the *Great wave*, considering its transverse localization is essential to any attempts to understand its origin and to propose plausible underlying physical mechanisms. It is also important to realize that the process of wave breaking itself is not confined to the simple case of extended surf waves on a beach, but occurs with all large-amplitude waves. The photographic evidence provided here of a breaking wave in the Southern Ocean is a striking example. Although it is not possible to make any unambiguous conclusions about the particular wave or waves that may have been seen by Hokusai to inspire his celebrated woodcut, we believe that directional focusing is a plausible mechanism that predicts wave characteristics highly similar to those of *The great wave off Kanagawa*. In terms of the artwork of the woodcut itself, highlighting the physics of the transverse localization of the *Great wave* provides room for unexpected optimism when interpreting the scene that is depicted. Transverse localization due to an effect such as directional focusing might suggest that the boats may not be in as much danger as usually believed. Is Hokusai really trying to highlight the skilful way in which the Japanese crews are navigating around the wave to avoid it breaking over them?

Our discussion also highlights the generality of wave localization phenomena in nature that can arise from both linear and nonlinear processes, although it is clearly difficult to differentiate which process may be dominant on the basis of field observation alone. Interestingly, this brings to mind a particular problem in the interpretation of water wave phenomena that dates back to the nineteenth century, and whose resolution in the twentieth century is linked to the very beginning of the field of nonlinear science itself. The nineteenth-century event of relevance here was John Scott Russell's 1834 observation of the ‘Great wave of translation’ on the Union Canal in Scotland.^17^ Although Scott Russell himself was convinced that what he had seen was a fundamentally new class of wave, this interpretation was contentious, and authorities at the time such as Stokes and Airy preferred not to accept the need for any new theoretical ideas beyond the notions of water wave theory at the time.^18,19^ It was only much later in the 1960s, with the development of techniques such as inverse scattering and numerical methods for solving nonlinear differential equations, that the full nonlinear theory of solitons provided the context with which to understand Scott Russell's observation completely.^20^

Ongoing research in the hydrodynamics community concerning the role of linear and nonlinear effects in rogue wave emergence continues this tradition of debate as to whether observations of novel waves in nature really require new theoretical descriptions.^9^ The field of rogue wave study today is of course in a much better position than Scott Russell was, in that the tools of advanced mathematics allow detailed simulations of a wide range of water wave propagation behaviour. However, even now we are very often in the situation where we must compare our numerical modelling with the laboratory of the ocean itself, where not all parameters are known or controlled. This serves to highlight the central role of observation above all, and the tremendous importance of talented observers (such as Hokusai) to capture the physics of the natural world.

## Notes

1 J. H. E. Cartwright and H. Nakamura, ‘What kind of a wave is Hokusai's *Great wave off Kanagawa*?’, *Notes Rec. R. Soc.* **63**, 119–135 (2009).

2 C. E. Synolakis, ‘Tsunami and seiche’, in *Earthquake engineering handbook* (ed. Wai-Fah Chen and Charles Scawthorn), ch. 9, pp. 9-1–9-90 (CRC Press, Boca Raton, 2003).

3 L. O'Brien, P. Christodoulides, E. Renzi, D. Dutykh and F. Dias, ‘The force of a tsunami on a wave energy converter’, in *Proc. Twenty-second International Offshore and Polar Engineering Conference, Rhodes, Greece, 17–22 June 2012* (ed. J. S. Chung, I. Langen, S. Y. Hong and S. J. Prinsenberg), pp. 538–544 (International Society of Offshore and Polar Engineers (ISOPE), 2012).

4 K. Kajiura, ‘Local behaviour of tsunamis’, in *Waves on water of variable depth* (*Lecture Notes in Physics* vol. 64) (ed. D. Provis and R. Radok), pp. 72–79 (Springer, Berlin, 1977).

5 C. Kharif and E. Pelinovsky, ‘Physical mechanisms of the rogue wave phenomenon’, *Eur. J. Mech. B/Fluids* **22**, 603–634 (2003).

7 C. Kharif, E. Pelinovsky and A. Slunyaev, *Rogue waves in the ocean* (*Advances in Geophysical and Environmental Mechanics and Mathematics*, vol. 14) (Springer, Berlin, 2009). (216 pages.)

8 R. C. T. Rainey, ‘Weak or strong nonlinearity: the vital issue’, *J. Engng Math.* **58**, 229–249 (2007).

9 Special issue on ‘Rogue waves—towards a unifying concept?’ *Eur. Phys. J. Spec. Top.* **185** (2010).

10 A. Chabchoub, N. P. Hoffmann and N. Akhmediev, ‘Rogue wave observation in a water wave tank’, *Phys. Rev. Lett.* **106**, 204502 (2011).

11 M. Onorato, A. R. Osborne and M. Serio, ‘Modulational instability in crossing sea states: a possible mechanism for the formation of freak waves’, *Phys Rev. Lett.* **13**, 014503 (2006).

12 H. Tamura, T. Waseda and Y. Miyazawa, ‘Freakish sea state and swell–windsea coupling: numerical study of the Suwa-Maru incident’, *Geophys. Res. Lett.* **36**, L01607 (2009).

13 M. L. Banner and D. H. Peregrine, ‘Wave breaking in deep water’, *Annu. Rev. Fluid Mech.* **25**, 373–397 (1993).

14 C. Fochesato, S. Grilli and F. Dias, ‘Numerical modeling of extreme rogue waves generated by directional energy focusing’, *Wave Motion* **44**, 395–416 (2007).

15 G. F. Clauss, ‘Dramas of the sea: episodic waves and their impact on offshore structures’, *Appl. Ocean Res.* **24**, 147–161 (2002).

16 I. Nikolkina and I. Didenkulova, ‘Rogue waves in 2006–2010’, *Nat. Hazards Earth Syst. Sci.* **11**, 2913–2924 (2011).

17 J. Scott Russell, ‘Report on waves’, in *Rep. Fourteenth Meeting of the British Association for the Advancement of Science, York, September 1844*, pp. 311–390, pl. XLVII–LVII (John Murray, London, 1845).

18 A. Darrigol, ‘The spirited horse, the engineer, and the mathematician: water waves in nineteenth-century hydrodynamics’, *Arch. Hist. Exact Sci.* **58**, 21–95 (2003).

19 A. D. D. Craik, ‘The origins of water wave theory’, *Annu. Rev. Fluid Mech.* **36**, 1–28 (2004).

20 A. V. Scott, *The nonlinear universe: chaos, emergence, life* (Springer, Berlin, 2007).

